# Perceptions and feelings of a French sample regarding lung cancer screening

**DOI:** 10.1186/s12889-023-17110-8

**Published:** 2023-11-24

**Authors:** Nicolas Darnaud, Jean-Eudes Mory, Pascal Romy, Jacques Berger, Karine Eve, Sophie Lantheaume

**Affiliations:** 1 Institut Supérieur Technologique Montplaisir, Valence, France; 2 Ramsay Santé Hôpital Privé Drôme Ardèche, Guilherand-Granges, France; 3 Laboratoire Inter-Universitaire de Psychologie, Personnalité, Cognition, Changement Social (LIP/PC2S), Université Grenoble Alpes, Grenoble, France

**Keywords:** Lung cancer, Screening, Perceptions, Participation, Smoking, Risk

## Abstract

**Introduction:**

This study investigated the perceptions and feelings of a French sample about the possible introduction of lung cancer screening.

**Methods:**

A total of 146 individuals, aged between 19- and 64-years, participated in this study conducted between November 2020 and January 2021. Participants were divided into three groups according to their smoking status: (i) active smokers (G1); former smokers (G2); and non-smokers (G3). Each individual completed an online questionnaire evaluating their perceptions and feelings about lung cancer, screening and scans.

**Results:**

Overall, a higher percentage of former smokers (47%) indicated a greater willingness to participate in lung cancer screening compared to active smokers (19%) and non-smokers (32%). Active smokers and former smokers reported anxiety about the development of lung cancer. Active smokers who wished to participate in screening reported a greater motivation to reduce their tobacco consumption. The perception of lung cancer risk had less influence than age and socio-economic category on the participation in screening. Finally, stigma did not appear to be a barrier to undergoing screening.

**Conclusion:**

Active and former smokers were generally optimistic about screening; however, active smokers showed less inclination to participate in screening compared to former smokers and non-smokers. Three main factors appeared to influence this participation: the perception of the risk of developing cancer, age and socio-economic category.

**Supplementary Information:**

The online version contains supplementary material available at 10.1186/s12889-023-17110-8.

## Patient or public contribution

All participants were free to participate and gave their informed consent before taking part in the study. The authors affirm that participants provided informed consent for publication of the results. The study conformed to the General Rules on Data Protection (Règlement Général sur la Protection des Données; RGPD) and the Data Protection Act (Loi Informatique et Libertés), and the data collected were strictly anonymized. The study was based on the principles and rules of the Rapport Belmont, the Code of Conduct of Researchers of Caverni and the Declaration of Helsinki.

## Introduction

Lung cancer is the third most common cancer in France and the leading cause of cancer-related death [1]. It is estimated that 46 363 new cases of lung cancer were diagnosed in 2018. The incidence of this disease decreased slightly in men between 1990 and 2018, while it increased in women due to a delayed uptake of smoking and slower cessation compared to men [2].

Several risk factors for lung cancer have been identified, but smoking remains the most important (in 90% of cases), making lung cancer a stereotyped and stigmatized disease. The literature reveals that this type of cancer is often more stigmatized than other diseases, because the development of lung cancer is closely associated with smoking, and patients are being held responsible for their condition [3]. This stigmatization may have important consequences on smokers, such as refusing medical visits, seeking them for benefits, or delaying their care [4, 5].

Currently, screening is organized for three types of cancer in France: breast cancer, colorectal cancer and cervical cancer. Several different barriers to screening have been found for these three types of cancer. The first is age, as seen in the *EDIFICE* study which reported a higher participation rate in colorectal cancer screening among individuals aged 70‒74 years than among those aged 50‒54 years [6]. The second is how often individuals consult their general practitioner (GP) or treating physician. For breast cancer, the National Institute for Prevention and Health Education (INPES) demonstrated a higher rate of adhesion to screening in individuals who had regular follow-up than for those who did not [7]. The third, is the fear of the disease [6]. When targeted individuals are faced with screening, their medical status shifts from a healthy state to a potentially pathological state [8]. This transition may be anxiogenic and expose the individual to various frightening perspectives or projections. As a consequence, some individuals prefer not to undergo screening faced with these negative thoughts. Another barrier is the involvement of GPs. A barometric study carried out by INCa revealed that one GP out of three does not systematically check whether their patients adhere to screening. However, this study showed that in 60–70% of cases of breast and colorectal cancer, patients considered the GP's role in screening indispensable [9–11]. Additionally, low levels of education and/or socio-economic category are significant factors. The *EDIFICE* study revealed lower participations rates (at least one test during life) and retention rates (one test in the last 2 years) among individuals facing social insecurity compared to those who are more socially secure [6].This can be explained by their attitude towards their own health, as some individuals view health as something to be treated rather than prevented. Furthermore, the *Haute Autorité de Santé* (HAS) reported that individuals in independent professions, such as shopkeepers, craftsmen and farmers, were less likely to participate in screening, possibly because health was not a concept anchored in their class and working life [9]. The limitations to organized screening (overdiagnosis, false-positives, etc.) are also a concern to some individuals [11]. In the case of cervical cancer, there is a lack of understanding about the disease and its screening [9].Finally, stigmatization is an obstacle to screening (particularly when individuals are considered to have played an active role in the onset of their disease, or when the disease is frightening) and is responsible for late reporting of symptoms, notably in the case of lung cancer [3, 4].

Today in France, there is no organized screening for lung cancer. Nevertheless, the HAS emphasizes the necessity to persist research in this area, especially through real-life experiments, and to intensify anti-smoking efforts. The HAS does not oppose early lung cancer screening to improve the chances of curing patients [12]. In fact, the HAS recognized its effectiveness since February 2022. A program led by The National Cancer Institute (INCa) is currently in progress; its results are expected by the end of 2024 or 2025. The screening examination for this type of cancer is a low-dose chest CT scan without injection, utilizing an imaging technique called computed tomography.

Several studies have demonstrated the benefits of screening a target population using low-dose thoracic CT scans [13–20]. Two of these stand out as being sufficiently large to detect any difference in lung cancer mortality with sufficient power: the randomized American National Lung Screening Trial (NSLT) [20] and the NELSON (Nederlands – Leuvens Longkanker Screenings Onderzoek) trial [18]. In 2011 and again in 2021, a group of French experts from various organizations including the *Intergroupe francophone de cancérologie thoracique* (IFCT, French-speaking thoracic oncology group), the *Société d'imagerie thoracique* (SIT, French Thoracic Imaging Society), representing the *Société française de radiologie* (SFR, French Society of Radiology), and the *Groupe d'oncologie de langue française* (GOLF, French-speaking oncology group), representing the *Société de pneumologie de langue française* (SPLF, French-speaking pneumology society), issued recommendations for individual lung cancer screening [21–23]. Individual screening is defined as the systematic proposal of screening, as described in the recommendations, by a physician to an eligible patient, outside any nationally organized program (Table 1).

**Table 1:** Inclusion, non-inclusion and exclusion criteria for individual lung cancer screening [22]

Inclusion criteria	Non-inclusion criteria	Exclusion criteria
Age between 50 and 74; and smoking > 10 cigarettes/d for > 30 years or > 15 cigarettes/d for more than 25 years; and active smoking or smoking cessation for ≤ 10 years or ≤ 15 years(OPTION); and willingness to undergo screening after informed consent; and willingness to help with smoking cessation	Inability to climb two flights of stairs without stopping; Weight ≥ 140 kg; History of a thoracic CT scan < 1 year old (excluding screening scans); History of bronchopulmonary cancer < 5 years old or currently under treatment; History of cancer being monitored by thoracic imaging; Severe co-morbidity contraindicating therapeutic possibilities or invasive thoracic diagnostic explorations; Current or recent respiratory symptoms suggestive of cancer (such as hemoptysis or recent pulmonary infection)	Duration of smoking cessation > 10 years (15 years optional); Age > 74 years after 3 scans (excluding studies); Occurrence of a non-inclusion criteria

The aim of this study was to investigate the perceptions and feelings towards the introduction of lung cancer screening among a French sample and to identify potentials barriers and harms associated with screening for individuals at risk in the future.

## Materials and methods

### Study design

This observational, descriptive study was carried out between November 2020 and January 2021. An online questionnaire was used to obtain information about the perceptions and feelings towards screening for lung cancer among the French general public.

The study conformed to the General Rules on Data Protection (Règlement Général sur la Protection des Données; RGPD) and the Data Protection Act (Loi Informatique et Libertés), and the data collected were strictly anonymized. The study was based on the principles and rules of the Rapport Belmont, the Code of Conduct of Researchers of Caverni and the Declaration of Helsinki.

### Study population

The study population was chosen to investigate the differences in perceptions and feelings among a sample of French people rather than being targeted at active smokers. There were no specific inclusion criteria and any adult who agreed to participate was accepted into the study. For the analysis, the study population was divided into three groups: active smokers, former smokers and non-smokers.

Active smokers were defined as individuals who smoked every day or occasionally but not daily, for example during social events. Former smokers were defined as someone who had smoked in the past but no longer smoked at the time of the study. A non-smoker was defined as a person who has smoked less than 100 cigarettes during his life [24]. Studies in sociology and psychology highlight the significant role that an individual’s social circle can play in their participation in screening. Some relationships help individuals maintain their health by ensuring they adhere to their responsabilities [25]. Furthermore, studies on mutations in lung cancer has revealed a noteworthy percentage of cases among non-smokers or individuals with minimal smoking history [26]. Thus, these two points encouraged us to include non-smokers in our study, in order to collect their perceptions and feelings on national lung cancer screening program as well.

### Tools

To assess perceptions and feelings regarding lung cancer, lung cancer screening, and scans, a questionnaire comprising both open-ended and closed-ended questions was developed specifically for this study. It was distributed via an online platform. On average, participants took approximately 15 min to complete the questionnaire.

Sociodemographic data including age, gender, marital status, presence of children at home, and socioeconomic category, were collected. For active smokers and former smokers, additional information was collected: age at which they started smoking, number of cigarettes smoked per day, and age at which smoking was stopped. Furthermore, the number of years of smoking was only collected for former smokers.

The questionnaire is provided in the Additional file 1: Appendices.

### Statistical analysis

In order to compare the three groups, quantitative data (closed-ended questions) were analyzed using SPSS.22© (Statistical Package for the Social Sciences). Qualitative data (open-ended questions) were treated manually, by an analysis of thematic content in order to reveal and explore perceptions and feelings of the participants towards lung cancer screening.

## Results

### Characteristics of the study population

A total of 146 individuals participated in this study, with a mean (SD) age of 36.5 ± 14.0 years. The characteristics of the three patients’ groups are summarized in Table 2.

**Table 2:** Characteristics of the study population according to sub-group

	**Active smokers (G1)** **(*n* = 42)**	**Former smokers (G2)** **(*n* = 30)**	**Non-smokers (G3)** **(*n* = 74)**
Age (years)			
Mean ± SD	35.9 ± 12.6	44.2 ± 12.7	33.8 ± 14.3
Gender (%)			
Male	38.1	53.3	35.1
Female	61.9	46.7	64.8
Marital status (%)			
Married	30.9	46.7	31.1
Single	52.4%	30	48.7
Co-habiting	9.5	3.3	4
Widowed	0	0	0
Not known	7.1	20	16.2
Children (%)			
No	50	30	44.6
Yes	47.6	66.7	35.1
Not known	2.4	3.3	20.3
Socio-economic category (%)			
Craftsperson, shopkeeper, business owner	4.8	0	0
Manager, higher intellectual profession*,* intermediate profession (teachers and healthcare workers)	38.1	53.4	41.9
Qualified employee	11.9	23.3	4
Laborer	7.1	3.3	2.7
Student	33.3	16.7	47.4
Not known	4.8	3.3	4
Age (years) at starting smoking			
Mean ± SD	16.3 ± 2.1	16.6 ± 10.0	
Age (years) at quitting smoking			
Mean ± SD		7.8 ± 8.5	
No. of years of smoking			
Mean ± SD		16.6 ± 10.0	
No. of cigarettes smoked/day			
Mean ± SD	7.7 ± 5.2	13.5 ± 8.1	

Subjects in G1 were significantly younger than those in G2 (*p* = 0.0033), while subjects in G2 were significantly older than those in G3 (*p* = 0.002). In G3, there were more individuals in the socio-economic categories of company managers, executives, higher intellectual professions, and students compared to the other categories (*p* = 0.012).

### Stigma associated with lung cancer

There was no significant difference in the perception that society had a critical view of lung cancer among the three groups. All three groups reported that this perception would not have any impact on their participation in the national screening program for lung cancer.

### Perceptions and feelings of lung cancer screening

#### Quantitative results

These results are summarized in Table 3.

**Table 3:** Responses to the questionnaire and differences according to the groups

	Active smokers (G1) (*n* = 42)	Former smokers (G2) (*n* = 30)	Non-smokers (G3) (*n* = 74)	*P*
Society's critical view of lung cancer				
Yes	31%	27%	39%	NS
No	60%	63%	47%	
No opinion	9%	10%	14%	
Awareness of national lung cancer screening program				
Yes	24%	20%	20%	NS
No	76%	77%	77%	
No opinion	0%	3%	3%	
Concerns about developing lung cancer				
Mean ± SD	1.90 ± 1.03	2.57 ± 1.33	2.66 ± 1.32	< 0.01
Concern that scans will detect cancer				
Mean ± SD	2.17 ± 1.27	2.40 ± 1.35	3.20 ± 1.24	< 0.001
Participation in national lung cancer screening program				
Yes	19%	47%	32%	0.068
Non	81%	53%	68%	
National lung cancer screening program feelings				
Joy	19%	0%	3%	< 0.01
Sadness	29%	23%	27%	NS
Fear	88%	73%	78%	NS
Anger	14%	7%	4%	NS
Disgust	12%	7%	1%	0.034
Surprise	29%	20%	23%	NS
Wish to participate in the national lung cancer screening program				
Yes	73%	77%	73%	
No	17%	20%	26%	
No opinion	9%	3%	1%	
Higher survival rate if national lung cancer screening program implemented				
Yes	95%	90%	96%	0.026
No	5%	0%	4%	
No opinion	0%	10%	0%	
Considering screening if detection accurate to 90%				
Yes	86%	67%	20%	NS
Non	14%	30%	77%	
No opinion	0%	3%	3%	
Considering screening if detection accurate to 70%				
Yes	72%	54%	47%	NS
No	26%	43%	49%	
No opinion	2%	3%	4%	

There was a trend toward a significant difference in potential participation in lung cancer screening among the three groups (*p* = 0.068). Active smokers appeared to be more reluctant to participate (19% wished to participate) compared to former smokers (47%) and non-smokers (32%).

A significant difference was observed among the three groups in their perceptions of lung cancer screening. Active smokers reported experiencing more disgust (*p* = 0.034), but also more pleasure (*p* < 0.01) when considering screening, as compared to former smokers and non-smokers. The majority of participants in all three groups reported feeling fear as the main emotion associated with national lung cancer screening program. Additionally, all three groups were in agreement that early screening improved the chances of survival (*p* = 0.026).

Anxiety related to the prospect of developing lung cancer exhibited significant differences among the three groups (*p* < 0.01). Active smokers expressed higher levels of anxiety about developing lung cancer compared to former smokers and non-smokers. The three groups also demonstrated significant differences in their concerns about whether scans would detect cancer (*p *< 0.001). Active smokers were more concerned than non-smokers, while former smokers also expressed greater concern compared to non-smokers.

Furthermore, age was significantly correlated with anxiety about developing lung cancer (*p* = 0.041). Older participants were more likely to report worry about developing cancer, particularly noticeable among active smokers (G1), where age was significantly associated with the perception that smoking had long-term health consequences (*p* = 0.002). Older participants were more inclined to perceive smoking as detrimental to their health. Additionally, for active smokers, age was linked to a reduced willingness to participate in lung cancer screening (*p *= 0.03); the older the participant, the less desire they had to engage in screening.

Socio-economic category had a marginally significant impact on the participation in screening (*p* = 0.053). Company managers, executive and higher intellectual professionals, intermediate professions (teachers, healthcare workers) and students were more likely to participate in screening than other participants.

Participation in screening had a significant impact on the desire of participants to reduce their tobacco consumption (*p* = 0.02); approximately two-thirds of active smokers reported that the introduction of screening would lead to a reduction in their tobacco consumption (Table 4). For G2 (former smokers), the belief that early detection of lung cancer would result in a favorable prognosis had a significantly positive effect on their willingness to participate in screening (*p* = 0.012). Similarly, the perception that scans would decrease the risk of death from lung cancer had a positive effect on the participation in screening (*p* = 0.041).

**Table 4:** Effect of screening on tobacco consumption among active smokers (G1)

Theme	Sub-theme	Citations
**Continue smoking (71%)**	Lack of motivation (20%)	*‘No desire to stop’*
	Pleasure of smoking (16%)	*‘Value it’*
	Presence of screening (16%)	*‘Frequent control’*
	Smoke little (12%)	*‘Occasionally’*
	Smoke less (8%)	*‘I smoke but less’*
	Not concerned (4%)	*‘Still young’*
**Smoke less (69%)**	Fear (20%)	*‘Fear of the result’*
	Self-preservation (16%)	*‘To avoid bad news’*
	To stop (12%)	*‘Envisage stopping’*
	Risk (8%)	*‘Due to the risks incurred’*
	To know (4%)	*‘Know the risk to react’*
**Stop smoking (23%)**	Fear (50%)	*‘Fear of cancer’*
	To know (13%)	*‘To know the truth in order to react’*
	Stop (13%)	*‘I will stop’*

## Qualitative results

**Fig. 1:**
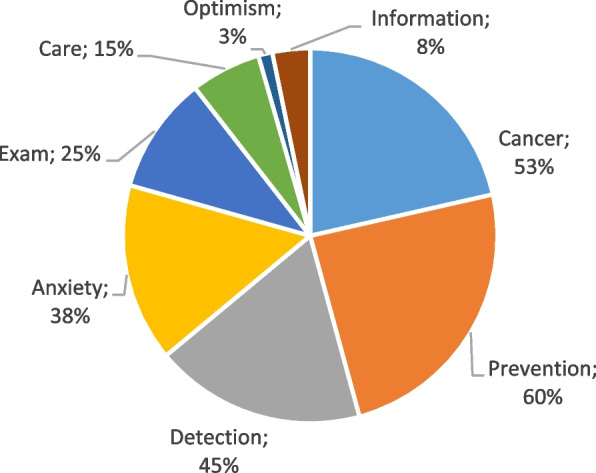
Perceptions about screening for lung cancer among active smokers (G1)

**Fig. 2:**
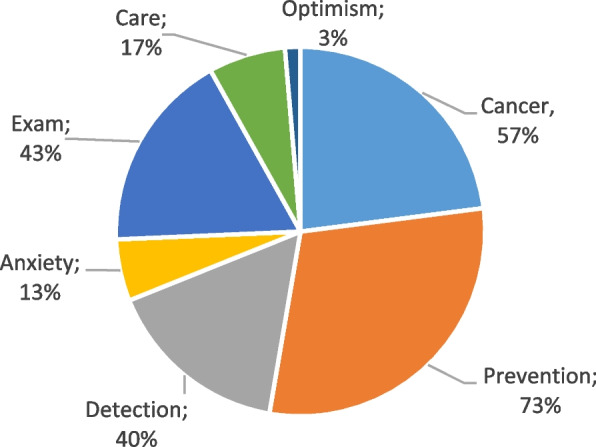
Perceptions about screening for lung cancer among former smokers (G2)

**Fig. 3:**
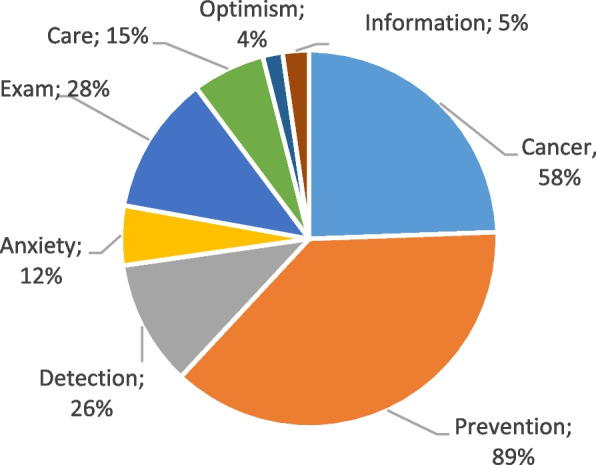
Perceptions about screening for lung cancer among non-smokers (G3)

When study participants were asked to provide the three terms that they most associated with the word ‘screening’ (Figs. 1, 2 and 3), ‘’*prevention’ ‘* and *‘’cancer*’ ‘ were the most frequently cited terms. Notably, active smokers (G1) also mentioned terms related to anxiety, ‘’*distress’*’ and ‘‘*worry*’’, in contrast to those in the other two groups. These results are summarized in Table 5.

**Table 5:** Perceived advantages and disadvantages of screening among the three groups

Themes		Sub-theme	*Citations*
**Advantages**	G1 (90%)	Prevention (58%)	*‘To remove the doubts’*
		Early diagnosis/Rapidity (46%)	*‘To take care of themselves more quickly.’*
		To know (22%)	*‘To know the state of their health’*
		Treatment/Care (24%)	*‘Cure’*
		Prognosis (8%)	*‘Better chance of cure’*
		Detection (7%)	*‘Detection’*
		Reassuring (3%)	*‘Reassure’*
	G2 (83%)	Early diagnosis/Rapidity (52%)	*‘Early detection’*
		Prevent (52%)	*‘Could save their life’*
		Treatment/Care (36%)	*‘Less intensive treatment’*
		To know (24%)	*‘To know the state of their health’*
		Detection (16%)	*‘Detection’*
		Reassuring (4%)	*‘Reassure’*
	G3 (93%)	Early diagnosis/Rapidity (55%)	*‘Early detection’*
		Prevent (35%)	*‘Avoidance’*
		Treatment/Care (32%)	*‘Less curative’*
		Detection (16%)	*‘Detection’*
		Reassuring (4%)	*‘Reassure’*
		To know (3%)	*‘To know’*
		Prognosis (3%)	*‘More chance of cure’*
**Disadvantages**	G1 (90%)	None (49%)	
		Fear/Anxiety (22%)	*‘Traumatic’*
		Constraints (22%)	*‘Need to travel’*
		To know (11%)	*‘Discover’*
		Unease (5%)	*‘Discouraged’*
		Examination (5%)	*‘Overdiagnosis’*
		Stress (5%)	*‘Stress’*
	G2 (87%)	None (50%)	
		Fear/Anxiety (23%)	*‘Discover the disease’*
		Stress (15%)	*‘Stress of the procedure’*
		Examination (8%)	*‘Irradiation’*
		Constraints (8%)	*‘Time’*
	G3 (88%)	None (48%)	
		Constraints (23%)	*‘Financial cost’*
		Fear/Anxiety (17%)	*‘Bad news’*
		Examination (11%)	*‘Radio-induced cancer’*
		Stress (8%)	*‘Stress of family members’*

Participants reported various benefits associated with participating in screening, with themes centered around 'early detection,' 'rapidity,' and 'prevention' being predominant in all three groups.. In addition, participants reported more benefits than harms associated with screening. Regarding differences between the three groups, both active and former smokers placed more importance ‘’on ‘learning’ and ‘knowledge’’ compared to non-smokers, while former smokers discussed ‘‘*treatment and care*’’ more frequently than active smokers.

Regarding the potential harms of screening, the majority of individuals in all three groups cited ‘’*none’ ‘.* Furthermore, the perceived harms associated with the examination were minimal. However, both active and former smokers mentioned ‘‘*fear’’* and *‘‘worry’’* more frequently than non-smokers. Active smokers also mentioned ‘‘*constraints*’’ linked to screening more often than former smokers, while, the latter reported experiencing more ‘‘*stress’’* than active smokers.

The majority of participants expressed that they would find ‘‘*early diagnosis*’’,’’*rapidity*’’ and ‘‘*knowing*’ reassuring. Both active smokers and former smokers, mentioned feeling ‘‘*reassured*’’ by screening, with a minority speaking of ‘‘*apprehension/fear*’’. However, these two groups differed in terms of the third theme that reassured them the most: active smokers emphasized the importance of ‘’*detection*’ ‘, while former smokers mentioned feeling ‘’more *at ease*’’.

Regarding the reasons for non-participation in screening, the majority of former smokers and non-smokers shared similar views. First, they did not consider themselves at risk, and second, they expressed concerns related to the limitations of the examination, such as ‘‘*irradiation*’’ or ‘‘*overdiagnosis*’’. A minority of active smokers did not feel concerned by screening. However, some active smokers mentioned that they chose not to participate because they preferred not to know. In addition, active smokers reported more fears related to the disease and anxiety compared to former smokers.

Finally, concerning motivation to stop smoking (Table 4), the majority (around 70%) of active smokers stated that screening would influence their tobacco consumption by reducing it, particularly if ‘‘*the diagnosis was positive*’’ or based on the ‘‘e*volution of the results*’*’*. However, some participants expressed that they did not wish to stop smoking or reduce their tobacco consumption, and a few even mentioned the possibility of increasing their consumption “*until something is detected”*, if screening were introduced, as they believed it would lead to earlier disease detection.

## Discussion

Several previous studies have highlighted the role of stigma as a barrier to lung cancer screening [13, 27]. In their research, Carter-Harris et al. identified distrust and stigma as perceived barriers to screening [27], while Tod et al*.* reported that blame and stigma contributed to delays in medical investigations [13]. However, our study, which included active smokers, former smokers and non-smokers, found that individuals who perceived a critical view of society towards lung cancer primarily in the form of stigma, did not believed it would impact their participation in screening. These results differ from those of previous studies and two factors may explain this difference: the axis of our study and the characteristics of our study population. In the study of Carter-Harris et al. [27], participants had a long history of smoking and perceived stigma as originating principally from younger healthcare providers. In contrast, our study linked stigma to a critical view of smoking by society in general. Additionally, Tod et al*.*’s study included participants who had received a diagnosis of lung cancer during the previous 6 months [13], while our study did not target individuals with lung cancer, and to our knowledge, none of our participants had lung cancer.

Finally, some of our participants reported constraints linked to screening, notably those related to timing and appointments. It is possible that these constraints may influence participation in screening, aligning with the findings of Carter-Harris et al. [27], who identified time constraints and scheduling conflicts as potential obstacles to lung cancer screening.

Overall, our qualitative results indicate that participants in all three groups reported more benefits than harms associated with screening. In fact, ‘*none*’ was the most common response when participants were asked about the harms of screening. Among the most frequently mentioned benefits were terms related to ‘*early diagnosis*’ and ‘*rapidity*’. Additionally, all three groups unanimously agreed that the chances of survival were significantly higher with early screening. These findings align with those of Carter-Harris et al*.*, who also reported the two main benefits found in our analyses: early detection of lung cancer and the potential for improved survival rater [27].

Our study identified two factors that encouraged former smokers to participate in screening: (i) those who believed that early detection of lung cancer leads to a better prognosis were more inclined to participate; and (ii) individuals who thought that scans would reduce the risk of death from lung cancer expressed their willingness to participate in screening. These findings suggest that former smokers generally hold a positive and optimistic image of screening. These results are consistent with the study of Cataldo, who found that participants who thought that early detection of lung cancer would lead to a better prognosis were more likely to accept screening [14].

However, it is worth noting that in our study both active smokers and former smokers expressed anxiety about the possibility of scans detecting lung cancer. This fear was also reported by more than 50% of the participants of Cataldo’s study [14]. Anxiety related to a cancer diagnosis could potentially act as a barrier to screening.

Furthermore, active smokers reported greater anxiety at the idea of developing lung cancer compared to non-smokers. This heightened anxiety may be attributed to their awareness of the increased risk associated with their smoking habits. Kummer et al*.* also demonstrated this phenomenon, highlighting that smoking status and perceived risk played a role in individuals’ attitudes towards lung cancer screening [28]. In addition, our study revealed a participation rate in screening of 19% among active smokers, in contrast to 47% among former smokers. This suggests a slight difference in the desire to participate in screening based on the smoking status. Former smokers displayed a greater inclination to participate, which contrast with the preferences of active smokers and non-smokers. These results contradict those of the study of Cataldo, which reported that the perception of disease risk was one of the predictive factors for participation in screening [14]. However, they align with the conclusions of Ali et al. [29], who found that individuals with a higher perception of lung cancer risk were less likely to participate in screening. Upon comparing the characteristics of their study population with ours, their conclusion could potentially be explained by two factors: age and tobacco consumption.

In this same study, older age was linked to non-participation in screening. Similarly, our study also demonstrated that age significantly influences participation in screening. Specifically, it was observed that the youngest participants (mean age: 33 years) were more inclined to undergo screening compared to their older counterparts (mean age: 47 years). Notably, the individuals surveyed in Ali et al.’s study were older (between 50- and 75 years) than those in our study [29]. This suggest that age could indeed be a potential predictive factor for screening participation.

These results can also be attributed to the level of tobacco consumption. For instance, in a study carried out among Afro-Americans, most participants believed they were at high risk of developing lung cancer. Surprisingly, 92.9% of them were willing to accept low-dose computed tomography scans [30], despite having an average daily smoking habit of nine cigarettes per day. In our study, we found a similar level of tobacco consumption among our participants, with a mean of eight cigarettes per day). Consequently, when comparing our results with those of Tseng et al. [30] and Ali et al. [29], it becomes evident that a lower perceived risk of developing lung cancer (linked to a low level of tobacco consumption) may impact participation in screening.

The results of our study also show that the desire to participate in screening among active smokers had a significant impact on their willingness to reduce their tobacco consumption. These findings contradict those of an American study conducted by Zeliadt et al. [31], where 49% of participants reported that screening reduced their motivation to stop smoking. The discrepancy in results may be attributed to differences in the study population. In the study of Zeliadt et al*.*, participants had a long history of smoking, averaging of 49 packet-years, 1 packet per day for 49 years or 2 packets a day for 25 years [31]. In contrast, in our study participants had a shorter average smoking history, not exceeding 20 years. Flynn et al*.* reported that long-term smokers might underestimate the impact of smoking on lung cancer risk and/or identify illusory counter-factors [32]. Other authors have found that some individuals believed that screening and the possibility of follow-up scans offered protection against lung cancer [31], while others estimated they could continue smoking until the results indicated signs of cancer in their lungs [30]. Our study also found that some individuals would reduce their tobacco consumption if the diagnosis was positive, some based on the of the evolution of the results, or even that their consumption would increase until something is detected. This latter approach is known as ‘Ostrich policy’, signifying the refusal to acknowledge danger.

Belonging to a specific socio-economic category also significantly influenced participation in screening. Managers, executives and higher intellectual professionals, intermediate professionals (such as healthcare workers and teachers), and students expressed a greater desire to participate in screening than other participants, including craftsmen, shop workers, qualified employees and factory workers. These findings align with two previous studies, which reported that individuals in highest socio-economic categories were more likely to participate in screening [29], and that there is a strong positive correlation between the highest socio-economic category and a positive response to an invitation to screening [33]. In the present study, this decrease in participation in lower socio-economic categories (such as independent professions) can be explained by a poor self-evaluation of health [33]. In these professions, health is not a concept anchored in their class and working life [9]. McRonald et al*.* also noted that lower socio-economic categories are at higher risk of developing the disease due to variations in tobacco use; therefore, individuals in these groups are less likely to accept screening [33]. To address this disparity, Quaife et al. proposed a more cost-effective and targeted invitation strategy such as inviting high-risk individuals, particularly smokers in lower socio-economic groups, to a pulmonary health assessment carried out by a nurse, aiming to improve engagement, in lung cancer screening [34].

The main strength of this study lies in the inclusion of three distinct groups, providing a comprehensive representation of attitudes towards lung cancer screening. Many previous studies have focused solely on active smokers, whereas mixed method-approach, combining quantitative and qualitative data, allowed us to identify specific indicators, whilst acknowledging the individuality of the respondents. However, it is important to note some limitations. The size of the sample may not fully represent the broader population. Additionally, a deeper exploration of the perception of cancer risk among active smokers could provide further insights into its impact on screening participation. Further research could extend this study by investigating perceptions toward lung cancer screening among GPs and pulmonologists, including any hesitations or potential barriers.

## Conclusion

This study reveals varying attitudes toward screening among individuals, including both active and former smokers, with an overall optimistic view of the process. However, both active and former smokers exhibit higher levels of fear and anxiety regarding lung cancer and its associated screening. Interestingly, the perception of lung cancer risk appears to have less influence on participation compared to age and socio-economic status. Notably, stigma does not seem to deter individuals from desiring to participate in screening. It is important to acknowledge that the study's limitations restrict the generalizability of these findings to the broader French population. Additionally, these results should be considered in the context of the screening eligibility criteria.

### Supplementary Information

**Additional file 1.**

## Data Availability

The datasets generated and/or analyzed during study are note publicly available but are available from the corresponding author on reasonable request.
