# Supplementary material for: Perceptions and feelings of a French sample regarding lung cancer screening
Source: BMC Public Health. 2023 Nov 24;23:2333. doi: 10.1186/s12889-023-17110-8 (PMC10675874; doi:10.1186/s12889-023-17110-8)
Supplement: Supplementary file 1 — Additional file 1. [file 12889_2023_17110_MOESM1_ESM.docx]

**Additional file 1: Appendices: Questionnaire on perceptions and feelings regarding lung cancer screening**

As a medical imaging student, I am carrying out a study into people's perceptions and feelings regarding national lung cancer screening program. This is an anonymous questionnaire. By agreeing to respond, you are consenting to participate freely and voluntarily in this study.

Part 1: Your profile

Question 1: What is your gender?

- Male
- Female

Question 2: What is your birth year?

Question 4: What is your marital status?

- Single
- Married
- Widowed
- With children
- Without children

Question 5: Employment status:

- Employed
- Retired
- Student

Question 3: Today, are you:

- A non-smoker
- An active smoker
- A former smoker

Question 4: Do you know anyone (family, friends) who has or has had lung cancer?

- Yes
- No

If so, do you think it can have an impact on your behavior?

- Yes
- No

If ‘yes’, in what way(s)?

Part 2: Lung cancer screening

Question 5: When you hear and/or read the word *‘screening’*, what terms do you associate it with? List 3 words.

Question 6: What do you see as advantages of national screening program (as seen in systematic screening for breast or colorectal cancer, for example)?

Question 7: Of these six emotions, which one(s) do you associate most closely with the concept of lung cancer screening?

- Joy
- Sadness
- Fear
- Anger
- Disgust
- Surprise

Question 8: Do you perceive negative representations of lung cancer in society?

- Yes
- No
- No opinion

If ‘yes’, what are the effects, if any?
Question 9: Have you ever heard of national screening program for lung cancer?

- Yes
- No
- No opinion

If yes, how did you find out and what was your reaction?

Question 10: Please indicate your agreement or disagreement with the following proposals regarding lung cancer representations and systematic screening by checking the respective boxes:

|  | Strongly agree  (1) | Agree  (2) | Tend to agree (3) | Disagree (4) | Strongly disagree  (5) |
| --- | --- | --- | --- | --- | --- |
| I am worried about developing lung cancer |  |  |  |  |  |
| When I hear the word ‘cancer’, I feel uncomfortable or it scares me |  |  |  |  |  |
| Smoking has long-term health consequences |  |  |  |  |  |
| Early detection of lung cancer leads to a good prognosis |  |  |  |  |  |
| I am worried about the scanner detecting cancer |  |  |  |  |  |

Question 11: Have you ever been screened for any other type of cancer?

- Yes
- No
- No opinion

If ‘yes’, how would you react if you had to make another one?

Question 12: If national screening program for lung cancer was to be implemented, and you met the screening criteria, would you get tested?

- Yes
- No
- No opinion

Why?
Question 13: In your opinion, would the chances of survival for people with lung cancer be higher if national lung cancer screening program were to be introduced in France?

- Yes
- No
- No opinion

Question 14: Do you believe that systemic lung cancer screening would affect your behavior towards people around you who smoke? (Question for non-smokers only)

- Yes
- No
- No opinion

Why?

Part 3: Low-dose scanner

Question 15: Please indicate your agreement or disagreement with the following proposals regarding lung cancer screening using a scanner by checking the respective boxes:

|  | Strongly agree (1) | Agree (2) | Tend to agree (3) | Disagree (4) | Strongly disagree  (5) |
| --- | --- | --- | --- | --- | --- |
| I find scans uncomfortable  / painful |  |  |  |  |  |
| I have no fear of undergoing a scan |  |  |  |  |  |
| I feel the same level of nervousness for scans as I do for any other imaging test |  |  |  |  |  |
| Radiation from scanners could lead to lung cancer |  |  |  |  |  |
| Scanning could reduce the risk of dying from lung cancer |  |  |  |  |  |
| A negative scan result will reduce the risk of developing lung cancer |  |  |  |  |  |

Question 16: If a scanner had 90% accuracy in detection, would you be open to undergoing this test?

- Yes
- No
- No opinion

Question 17: If a scanner had 70 % accuracy in detection, would you be open to undergoing this test?

- Yes
- No
- No opinion

Thank you for participating
